# Effects of short-term exposure to low doses of bisphenol A on cellular senescence in the adult rat kidney

**DOI:** 10.1007/s00418-022-02178-x

**Published:** 2023-01-09

**Authors:** Paula Nuñez, Juan Arguelles, Carmen Perillan

**Affiliations:** Departamento de Biología Funcional, Área de Fisiología, Facultad de Medicina y Ciencias de la Salud, Universidad de Oviedo, Julián Claveria s/n, CP:33006 Oviedo, Asturias Spain

**Keywords:** Aging, Endocrine disruption, Kidney, Biomarker, Lipofuscin, IGF-1

## Abstract

Bisphenol A (BPA) is one of the primary chemicals produced by volume worldwide. Extensive literature has raised many concerns about its possible involvement in the pathogenesis of kidney diseases, but its contribution has not been extensively studied. During cellular senescence, the interference of lipofuscin with cellular functions promotes further senescence, causing cellular malfunction. Insulin-like growth factor-1 (IGF-1) plays an important protective role in the setting of kidney injury. The goal of the present work was to evaluate the effects of short-term treatment with low doses of BPA on cellular senescence in adult rat kidneys. Male Wistar rats were injected with vehicle (CONTROL group) or 50 or 500 μg/kg/day of BPA for 1 week (BPA50 and BPA500 groups, respectively). The kidneys were fixed in 4% buffered formaldehyde and embedded in paraffin. Immunohistochemical analyses were performed, and an immunoreactive score (IRS) was calculated. Lipofuscin autofluorescence was used for the study of cellular senescence. The renal cortex showed diffuse autofluorescent lipofuscin signal in the proximal convoluted tubules (PCTs) of males in the BPA50-treated (weak intensity) and BPA500-treated (strong intensity) groups, but not in CONTROL males. Labeling of cortical PCTs with anti-IGF-1 antibodies showed an IRS of 0 in the CONTROL group, but IRSs of 4 and 6 in the BPA50- and BPA500-treated groups, respectively. The present results suggest that low, “safe” doses of BPA induce renal injury, as measured by histological signs of renal changes, increased cellular senescence, and activation of cellular repair systems in PCTs.

**Supplementary Information:**

The online version contains supplementary material available at 10.1007/s00418-022-02178-x.

## Introduction

Bisphenol A (BPA) is one of the main volume chemicals produced worldwide. BPA is a “xenoestrogen” that has weak estrogenic activity due to binding to estrogen receptors and disturbing estrogen metabolism (Varticovski et al. 2022). Extensive literature has raised many concerns about its possible implications in the origin of some chronic diseases such as kidney diseases (Moreno-Gómez-Toledano et al. 2021). Physiologically established pharmacokinetic models suggest that renal tubular reabsorption of BPA conjugates contribute to serum BPA levels, but the contribution of this pathway to renal injury has not been deeply studied (Habeeb et al. 2022). Proximal tubule cells (PTCs) in renal cortex, which reabsorb many filtered molecules, are the primary site of kidney injury associated with nephrotoxicity (Treuting et al. 2018). Tissue markers associated with senescence are increased in injured and aging rodent kidneys (Luo et al. 2018; Docherty et al. 2020).

Cellular senescence is a permanent state of cell cycle arrest that promotes tissue remodeling during development and after injury. Senescent cells remain viable but show altered morphology, greater heterogeneity, developed hypertrophy, expression of SA-β-gal, accumulation of lipofuscin granules, and lack of response to mitogenic stimuli. Interactions of lipofuscin with cellular functions are able to increase its rate of formation, resulting in a vicious cycle, causing cellular malfunction and death (Di Guardo 2015). The evidence so far from in vitro and in vivo studies suggests that cellular senescence acts as a tumor barrier, whereas it contributes to the processes of tissue aging and age-related diseases (Rodier and Campisi 2011; Kowald et al. 2020). Actively proliferating cells efficiently dilute lipofuscin during successive divisions (Terman 2001). However, when proliferation of normally mitotic active cells is inhibited by, for example, growth factor starvation or confluency, lipofuscin starts to accumulate. It is known that tubular epithelial cells that survive damage secrete growth factors are involved in kidney repair mechanisms (Terman and Brunk 1998).

Insulin-like growth factor 1 (IGF-1) is a major regulator of postnatal somatic growth, mediating many of the effects of growth hormone. Circulating levels of IGF-1 reach their peak at mid-teen years and then decline with age (Allard and Duan 2018). When the concentrations of IGF-1 are high, the signal is mitogenic and anti-apoptotic (Valentinis and Baserga 2001). Apoptosis is a form of programmed cell death that results in the orderly and efficient removal of damaged cells. IGF-1 plays a protective role against apoptosis and regulation of cell growth, which facilitates recovery from acute kidney injury (AKI) (Peruzzi et al. 1999). Experimental evidence supports a pathogenic role for apoptosis in AKI. Proximal tubule epithelial cells are highly susceptible to apoptosis, and injury at this site contributes to organ failure (Havasi and Borkan 2011).

Many clinical and epidemiological studies have described the recurrent exposure of the general population to BPA as well as its harmful effects, but its contribution to renal injury has not been studied sufficiently. The goal of the present work was evaluated the effect of short-term treatment with low doses of BPA, similar to population levels, on cellular senescence in the adult Wistar rat kidney.

## Material and methods

This study was performed in accordance with guidelines established by the Institutional Animal Care and Use Committee at the University of Oviedo (PROE-15/2016). Rats were obtained from the Vivarium Unit of the University of Oviedo. Adult male Wistar rats (350–400 g, aged 4 months) were housed individually under standard conditions at 22 ± 3 °C with light/dark periods of 12 h and a minimum relative humidity of 40%. Rats were maintained on 2014 Teklad Global 14% protein rodent maintenance diet (Harlan Laboratories, Barcelona, Spain), which does not contain alfalfa or soybean meal (chow diet). The composition of the diet was as follows: calories from protein, 18%; calories from fat, 11%; calories from carbohydrates, 71%; energy content, 2.9 kcal/g. Rats were acclimatized to their housing conditions for at least 15 days.

BPA (Sigma, cat. number 239658) was dissolved in tocopherol-stripped corn oil (Panreac Química, cat. number 8001307) and administered subcutaneously during the early light phase for 7 days. Male Wistar rats were subcutaneously injected with vehicle (tocopherol-stripped corn oil, CONTROL group), or 50 or 500 μg/kg/day of BPA for 1 week (BPA50 and BPA500 groups, respectively). The sample size was five to six animals per group.

Rats were sacrificed under deep anesthesia (0.5 mL of sodium pentobarbital), blood samples were collected by cardiac puncture and then rats were immediately perfused via the left ventricle. One capillary tube was filled for hematocrit determination. Blood samples were centrifuged and serum was stored at −20 °C. Serum osmolality was determined using a Wescor 5100C Osmometer. The kidneys were removed and fixed (4% paraformaldehyde in 0.1 M phosphate buffer, pH 7.4) for 4 h. Next, they were washed in running water and incubated in 70% ethanol until processing (Nuñez et al. 2015). The kidneys were then embedded in paraffin, serial sectioned at a thickness of 5 μm, and stained with periodic acid–Schiff (PAS) stain and anti-insulin-like growth factor-1 (IGF-1) antibodies (1:100, Santa Cruz Biotechnology, cat. number sc-713). The Dako EnVision+ System kit (DAKO Corporation, cat. number K4003) was used according to the manufacturer’s instructions. The extent of renal damage on each slide was evaluated in several fields (Olympus BX53 microscope with cellSens image analysis software) with semi-quantitative histological and immunohistochemical analyses, and an immunoreactive score (IRS) was calculated. An average of ten light microscopic fields (with one or two glomeruli per field) per section was evaluated using a 20× objective.

We visualized green autofluorescence from lipofuscin (emission wavelength 450–490 nm) in 1 µm optical sections by confocal microscopy (Espectral Leica TCS SP8 X). This confocal laser scanning microscope is a spectral confocal microscope with an acousto-optical tunable filter, an acousto-optical beam splitter, and a spectral detector. It does not use a camera; images are serially constructed on the basis of the output from a photomultiplier tube. The objective lens specifications were: HC PL APO 40×/1.30 OIL PH3 CS2; magnification, 40×; numerical aperture, 1.30; spherical aberration (three to four colors); chromatic aberration (four to five colors); field curvature; cover glass, 0.17. The acquisition software was Leica Application Suite X version 1.8.1.

The semi-quantitative analysis of the stained sections was performed using a modified IRS originally developed by Remmele and Stegner (1987). The evaluation was based on the semi-quantitative immunoreactive, which was calculated by multiplication of optical staining intensity (graded as 0, none; 1, weak; 2, moderate; and 3, strong staining) and the percentage of positively stained cells (0, no staining; 1, ≤ 10% of the cells; 2, 11–50% of the cells; 3, 51–80% of the cells; and 4, ≥ 81% of the cells). In our study, the modified IRS evaluated the grade of staining intensity as well as the fraction of cells in each intensity category. The predominant intensity grade was used and the IRS (range 0–12) was adapted to an additional 4-point scale derived from the latter score (Supplementary Table 1) (McCarty et al. 1985; Kaemmerer et al. 2012; Naipal et al. 2015).

A widely accepted internationally standardized nomenclature for urinary tract lesions in laboratory animals was used in the present study to decrease confusion among regulatory and scientific research organizations in different countries, and provide a common language with which to facilitate and enrich international exchanges of information among toxicologists and pathologists (Frazier et al. 2012).

The data were analyzed using the Statistical Package SPSS 22.0 (IBM Corp., Armonk, NY, USA). We used the Kruskal–Wallis test to assess differences between treatment groups. Results were considered significant at *p* < 0.05. Data are shown as mean ± SEM.

## Results and discussion

Senescent cells accumulate in the kidney in three general settings: with age, with any insult causing acute kidney injury, and in chronic kidney disease. In each case, higher levels of senescent cells are associated with worsened kidney function and outcomes (Docherty et al. 2020). BPA is a ubiquitous environmental toxin; it is detectable in the urine samples of most adults and children because it is primarily removed by the kidneys (Doerge et al. 2010). Some reports have demonstrated that serum BPA levels are negatively correlated with annual changes in estimated glomerular filtration rates, identifying BPA as a renal damage biomarker (Azzouz et al. 2016). Previous studies have also shown that injection of 25 or 50 mg/kg/day of BPA resulted in albuminuria and podocytopenia. Although the exact cause of BPA-induced albuminuria is unclear, it may arise from oxidative stress-induced endothelial dysfunction.

In vitro studies have demonstrated that BPA causes mitochondrial injury, oxidative stress, and apoptotic death in tubular cells. However, in vitro models may not account for the interactions between cells and biochemical processes that occur during BPA metabolism in vivo (Priego et al. 2021). Previous work has shown that BPA participates actively in mechanisms of accelerated cell aging. For example, administration of toxicologic doses of BPA (e.g., 21.2 or 120 mg/kg/day, i.p. for 5 days/week) affected the vascular endothelium and promoted cardiovascular diseases in mouse models (Moreno-Gómez-Toledano et al. 2021; Priego et al. 2021). Several in vivo toxicity studies have also indicated that BPA exposure displays an inverted-U-shaped dose–response curve (a hormetic dose–response curve), often with no responses at high exposure levels (Lagarde et al. 2015). In 1993, the US Environmental Protection Agency defined the lowest observed adverse-effect level for BPA as 50 mg/kg/day and the “safe dose” as 50 μg/kg/day. However, the daily BPA intake of adult humans was found to be approximately 500 μg/kg/day (Taylor et al. 2011). Thus, the aim of the present work was to evaluate the effect of short-term treatment with low doses of BPA (50 or 500 μg of BPA/kg/day)—similar to the exposure levels of the human population—on cellular senescence in the adult rat kidney.

### Lipofuscin

Deposits of lipofuscin autofluorescence in proximal tubule cells (PTCs) of the renal cortex have been established as a biomarker for premature stress-induced senescence in the kidney (Georgakopoulou et al. 2013). Previous studies have shown that lipofuscin is a senescence biomarker comparable to senescence-associated β-galactosidase (SA-β-gal) activity. Some features of senescence in the aging kidney, such as the appearance of SA-β-gal or lipofuscin, are present even without morphologic changes, suggesting that some aspects of cell senescence are common in the aging kidney (Yang and Fogo 2010). Lipofuscin is a waste product that originates from a variety of intracellular structures and accumulates in the lysosome. Although lysosomal degradation occurs in all cells, only post-mitotic and slowly dividing cells accumulate lipofuscin. Hence, lipofuscin accumulation within post-mitotic cells is a hallmark of aging.

**Fig. 1:**
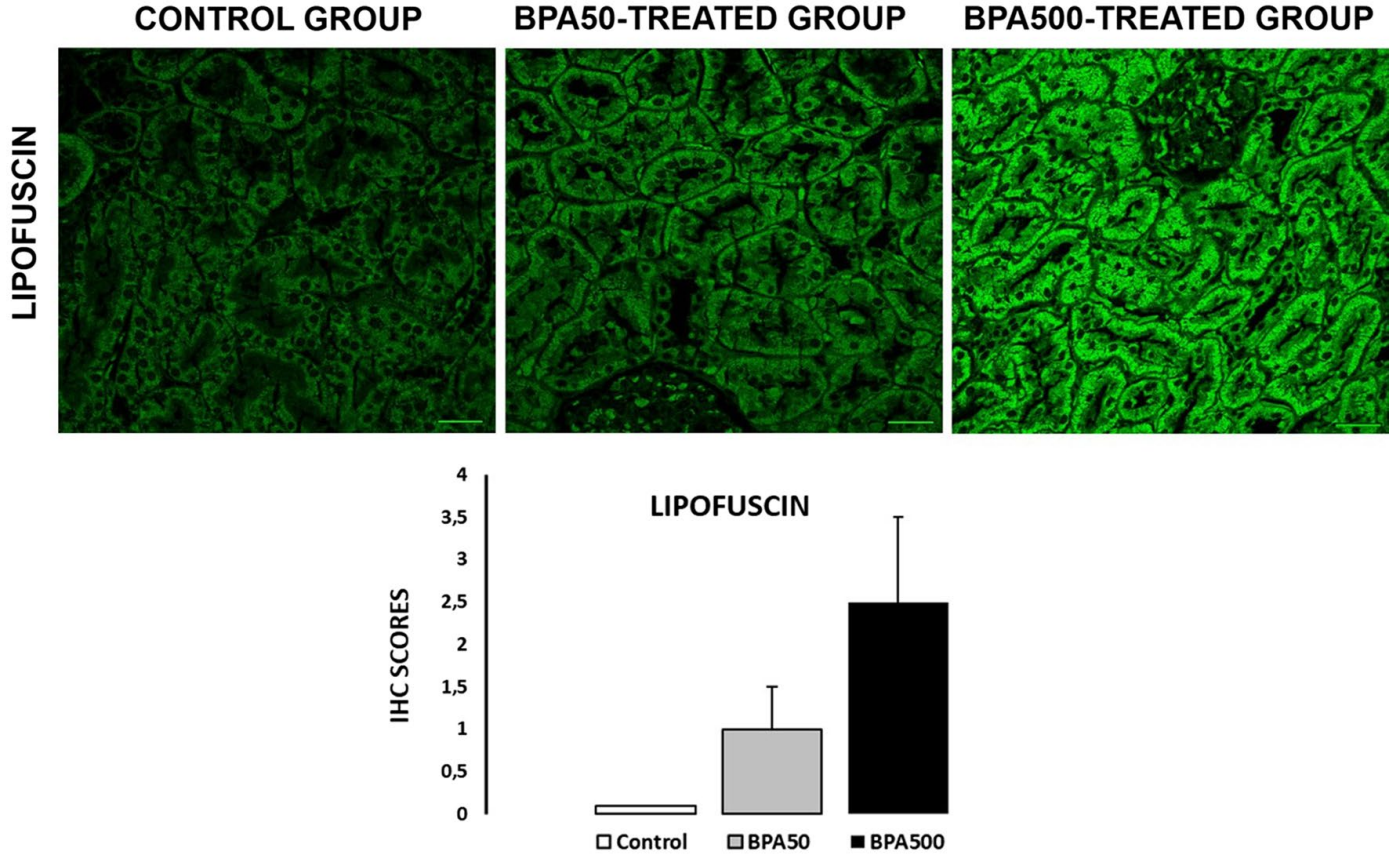
The immunoreactive score (IRS) of immunohistochemistry for the lipofuscin of CONTROL and BPA-TREATED GROUPS. Values are expressed as mean ± SEM. The sample size was five to six animals per group. Scale bar: 30 μm

In the present study, the PTCs in the renal cortex of the BPA-TREATED group exhibited diffuse cytosolic lipofuscin autofluorescence, whereas the CONTROL group did not (Fig. 1). Specifically, the kidney sections from the CONTROL group were negative for green lipofuscin autofluorescence (IRS: 0; Fig. 1), those from the BPA50-TREATED group showed mild green lipofuscin autofluorescence (IRS: 3; percentage of positive cells: 3; intensity of staining: 1; Fig. 1), and those from the BPA500-TREATED group revealed strong green lipofuscin autofluorescence (IRS: 6; percentage of positive cells: 2; intensity of staining: 3). No significant differences in lipofuscin IRSs were observed among the groups (*p* > 0.05; Fig. 1); however, IRSs tended to be higher in the BPA-TREATED groups. Lipofuscin increases with age in human kidneys and is present in tubular cells but not glomeruli. The distribution of lipofuscin and its increase in aged kidneys (Melk et al. 2004) are similar to what we observed in rat kidneys exposed to BPA. Although cells constantly recycle damaged components, the proportion of poorly functioning structures increases with age, particularly in post-mitotic cells, suggesting that the recycling machinery is imperfect (Glaumann et al. 1981). Our results suggest that low doses of BPA induced cellular senescence in the kidney and could play an important role in the development of kidney complications during aging.

### Insulin-like growth factor-1

Tubular epithelial cells that survive damage, secrete growth factors that can interact with resident cells such as renal and extrarenal stem cells, accelerating tubular repair mechanisms. It has a remarkable capacity for morphogenic regeneration after severe toxic or ischemic aggression (Anglani et al. 2004). The IGF system is expressed in a complicated manner within the kidney and has profound effects on kidney growth, structure, and function.

**Fig. 2:**
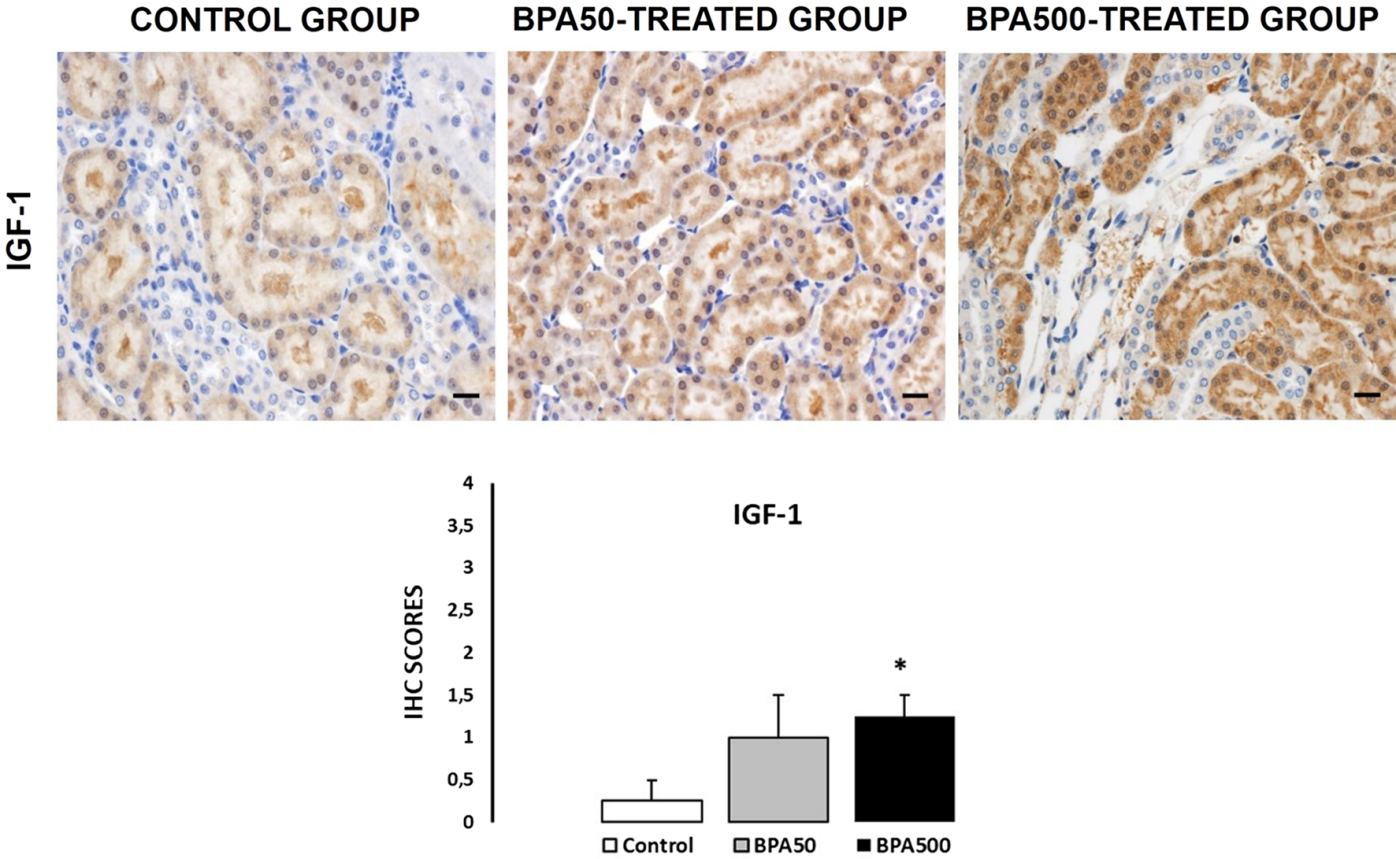
The immunoreactive score (IRS) of immunohistochemistry for insulin-like growth factor 1 (IGF-1) of CONTROL and BPA-TREATED groups. Values are expressed as mean ± SEM. *p < 0.05 versus CONTROL group. The sample size was five to six animals per group. Scale bar: 20 μm

IGF-1 staining revealed negative cytosolic expression in the cortical PTCs of animals in the CONTROL group, moderate expression in the BPA50-TREATED group, and strong expression in the BPA500-TREATED group (Fig. 2). Specifically, IGF-1 expression was negative (IRS: 1; percentage of positive cells: 1; intensity of staining: 1) in the CONTROL group, moderate in the BPA50-TREATED group (IRS: 4; percentage of positive cells: 2; intensity of staining: 2; Fig. 2), and strong in the BPA500-TREATED group (IRS classification: 9; percentage of positive cells: 3; intensity of staining: 3; Fig. 2). We observed statistical differences in the IRSs of BPA500-TREATED and CONTROL groups (*p* < 0.05; Fig. 2). These results suggest that IGF-1 may play an important role in protecting the kidney from BPA-induced damage. Other studies using animal models of acute kidney injury have been performed by administering growth factors such as IGF-1. These studies reported reduced mortality due to the restoration and normalization of kidney function (Hammerman and Miller 1994). In fact, it is well accepted that tubular epithelial cells that survive damage secrete growth factors involved in kidney repair mechanisms. This system can have several mechanisms of action, including autocrine (the kidney cells themselves secrete growth factors), paracrine (renal and bone marrow stem cells secrete growth factors), and endocrine (soluble factors are found in the circulation) (Flaquer et al. 2010).

### Kidney histopathology

**Fig. 3:**
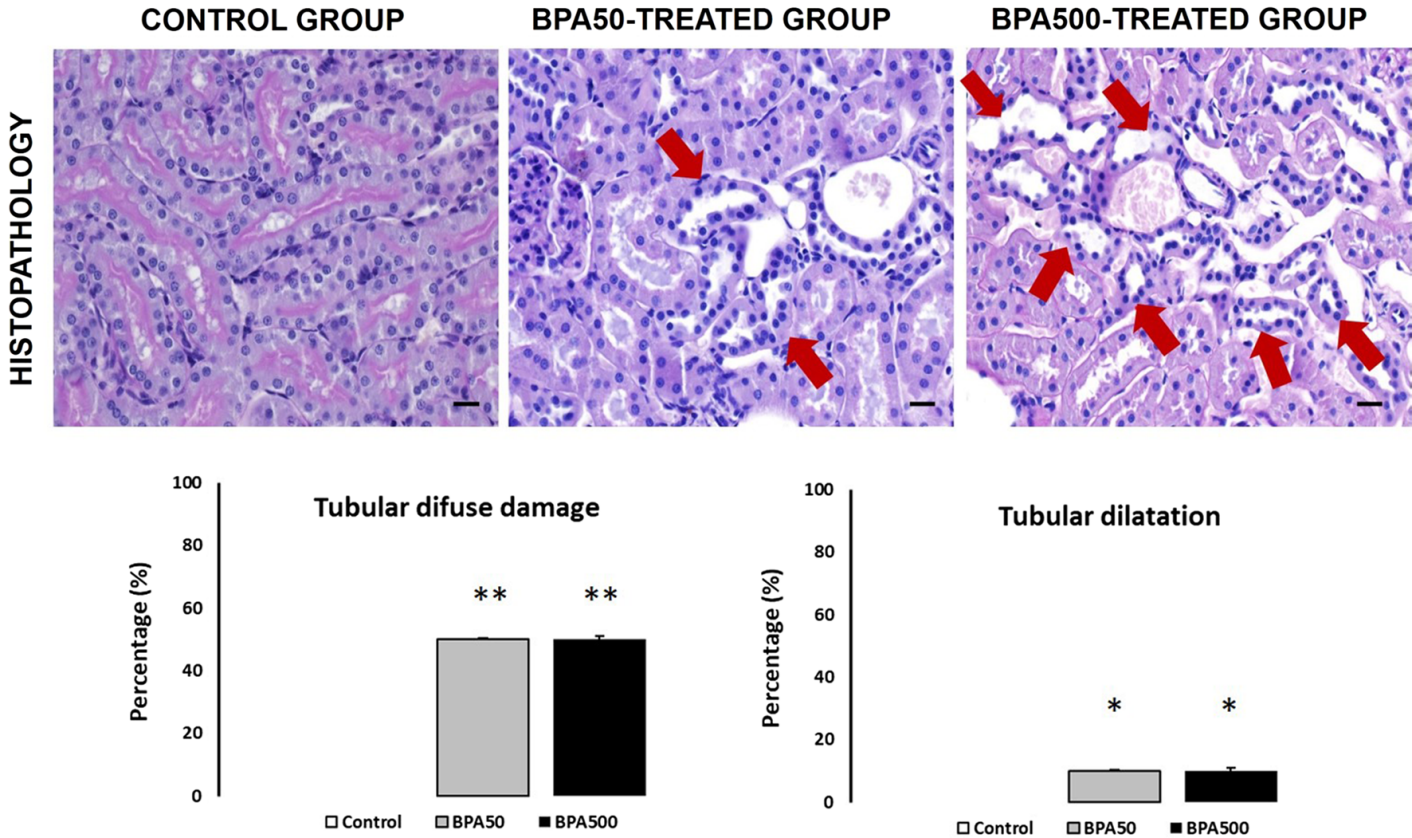
Representative micrographs of PAS stained cortical kidneys (proximal tubules) sections from CONTROL and BPA-TREATED groups were examined. Red arrow: tubular dilatation distension attenuated epithelial lining. Tubular/general lesions score (performed on ten-field  ×20 microphotographs of five slides of different renal heights, containing one to two glomeruli per photograph/field): 0: normal, < 10% tubular damage and 26–50% tubular damage. Scale bar: 20 μm. *p < 0.05 or **p < 0.01 versus control group

PTCs are energy-demanding cells sensitive to energy disruption, and loss of PTCs contributes to chronic kidney progression. Localization of the lesion to a specific segment within the kidney may be of great value in helping to determine the mechanism underlying xenobiotic-induced lesions in rodents (Treuting et al. 2018). The kidney is a common target organ for therapeutic and diagnostic agents. Renal injury may occur as a result of direct effects on tubules or glomeruli or indirectly via altered hemodynamics (Frazier et al. 2012). Representative micrographs of PAS-stained cortical kidney sections (proximal tubules) from the CONTROL and BPA-TREATED groups were examined (Fig. 3). Histopathological signs of acute epithelial cell injury were observed, including diffuse damage, involving less than 50% of the proximal tubules studied (*p* < 0.01), and tubular dilation, distension, and attenuated epithelial linings involving less than 10% of the tubules studied in the BPA-TREATED groups (*p* < 0.05; Fig. 3). Atrophic tubules showed flat, simple epithelial linings with thickened and wrinkled tubular basement membranes (Grgic et al. 2012). Tubular dilation most often accompanies other forms of renal damage (e.g., necrosis or degeneration) (Schetz et al. 2005). The pathogenesis of tubule dilation has been linked to tubular stasis, excessive renal hemodynamic changes, or electrolyte and water loss (Lameire 2005). However, these damages appear in a very small percentage (< 10%) in the BPA-treated groups. This kidney histopathology could contribute in part to the development of kidney problems in aging when cellular repair systems lose efficiency. It is known that cellular senescence is a beneficial compensatory response to damage that becomes deleterious and accelerates aging when tissues exhaust their regenerative capacity (Lopez-Otin et al. 2013).

**Fig. 4:**
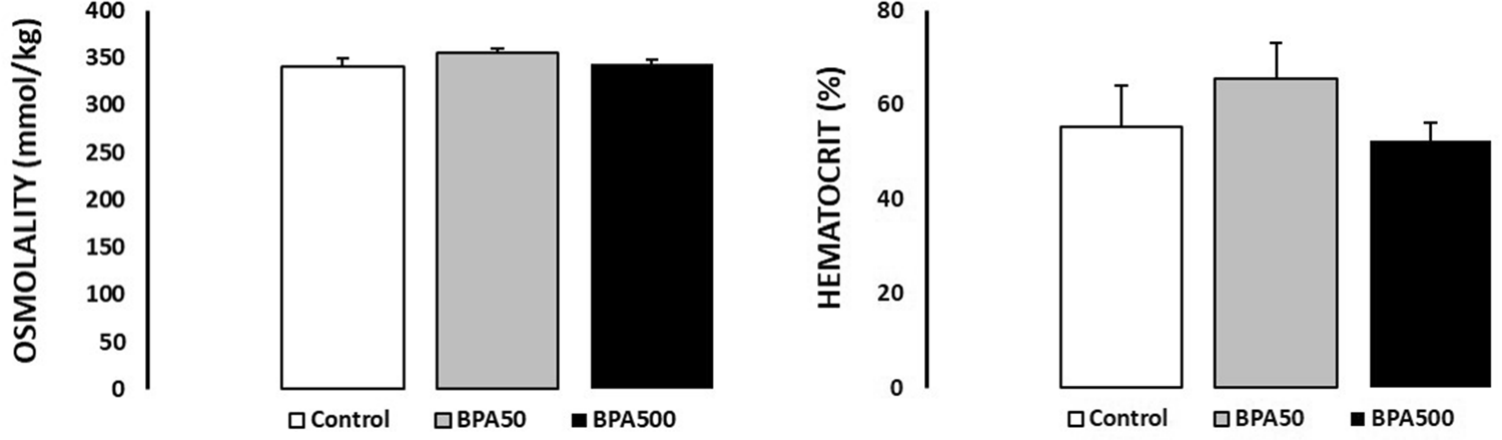
The serum osmolality (mmol/kg) and hematocrit (%) of CONTROL and BPA-TREATED groups. Values are expressed as mean ± SEM. There were no significant BPA effects on serum osmolality and hematocrit. The sample size was five to six animals per group

Serum osmolality is the serum concentration of ions and elements dissolved in body fluid to reflect the body fluid balance and renal function. Serum osmolality is a useful and valuable indicator to predict a kidney injury (Yang et al. 2021). A decrease in hematocrit is obvious even among patients with mild to moderate renal insufficiency (Hsu et al. 2001). No significant differences were observed in serum osmolality and hematocrit between CONTROL and BPA-TREATED groups (Fig. 4). A recent study showed that animals exposed to very low doses of BPA (0.5, 2, 4, 50, and 100 μg/kg BW/day) presented kidney alterations, such as increased urea and creatinine levels with respect to the controls, but these values are within the normal ranges; they did not produce clinical signs (Molina-López et al. 2022). In another experiment, mice exposed to a low dose of BPA (25 μg/kg BW/day) showed decreased urine volume, but no change in creatinine or plasma clearance was detected (Esplugas et al. 2018). One study in rats (Kobroob et al. 2021) and another in mice (Olea-Herrero et al. 2014) demonstrated that at least chronic exposure to 50 mg/kg BW/day is necessary to see changes in renal function (for example, an increased serum or urine creatinine or total urine proteins) and suggested that the renal functional impairment observed may lie in the damaging action of BPA on the kidney glomeruli. In the present study, the BPA-treated groups did not show glomerular damages.

## Conclusions

We found for the first time that the green autofluorescence from lipofuscin is a useful biomarker to improve the evaluation of cell senescence and monitor the responses to an endocrine disruptor in the kidney. The present results suggest that low “safe” doses of BPA induce signs of renal injury, including renal histological changes, increased cellular senescence, and activation of cellular repair systems in cortical PTCs. Further studies are needed to clarify the potential role of BPA in the pathogenesis and progression of renal diseases and aging.

## Supplementary Information

Below is the link to the electronic supplementary material.

Supplementary file1 Table 1. Immunoreactivity scoring system (IRS) (DOCX 34 KB)

## Data Availability

Data that support the findings of this study are available from the corresponding author upon reasonable request.
