# Supplementary figures and images for: Effects of short-term exposure to low doses of bisphenol A on cellular senescence in the adult rat kidney
Source: Histochem Cell Biol. 2023 Jan 9;159(5):453–60. doi: 10.1007/s00418-022-02178-x (PMC10192151; doi:10.1007/s00418-022-02178-x)

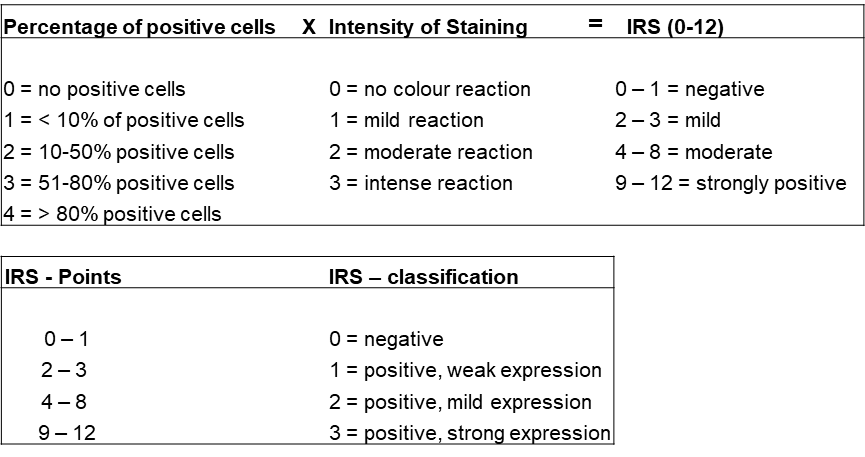

Supplement: Supplementary file 1 — Supplementary file1 Table 1. Immunoreactivity scoring system (IRS) (DOCX 34 KB) [file 418_2022_2178_MOESM1_ESM.docx]
